# Working behaviors and the risk of sensorineural hearing loss: A large cohort study

**DOI:** 10.5271/sjweh.4209

**Published:** 2025-03-01

**Authors:** Wendu Pang, Yao Song, Jun Xie, Xiaohong Yan, Yaxin Luo, PMH,, Ke Qiu, Yufang Rao, Di Deng, Minzi Mao, Junhong Li, Danni Cheng, Wei Xu, Jianjun Ren, Yu Zhao

**Affiliations:** 1 Department of Oto-Rhino-Laryngology and National Clinical Research Center for Geriatrics, West China Hospital, Sichuan University, Chengdu, China.; 2 Information Technology Center, West China Hospital of Sichuan University, Chengdu, China.; 3 Information Technology Center, Sanya People’s Hospital, Sanya, China.; 4 MRC Integrative Epidemiology Unit, Bristol Medical School, University of Bristol, Oakfield House, Oakfield Grove, Bristol, United Kingdom.; 5 Department of Biostatistics, Princess Margaret Cancer Centre and Dalla Lana School of Public Health, Toronto, Ontario, Canada.; 6 West China Biomedical Big Data Center, West China Hospital, Sichuan University, Chengdu, China.; 7 Department of Oto-Rhino-Laryngology, Langzhong People’s Hospital, Langzhong, China.

**Keywords:** night shift work, physically demanding work, **s**hift work

## Abstract

**Objectives:**

This study aimed to investigate the association between working behaviors and sensorineural hearing loss (SNHL).

**Methods:**

A cross-sectional analysis was conducted (N=90 286) to assess the association between working behaviors (including shift work, night shift work and physically demanding work) and the occurrence (yes/no), laterality (unilateral/bilateral), and severity (mild/severe) of SNHL. A prospective analysis was conducted to explore the association between new-onset SNHL and working behaviors (N=8341). Multivariable logistic regression and Cox regression models were performed. Subgroup analyses were further carried out, stratified by age, sex, and chronotype. Furthermore, a polygenic risk score (PRS) was calculated to assess the influence of genetic susceptibility on the relationship.

**Results:**

Cross-sectional analysis indicated that shift work, night shift work and physically demanding work were all associated with an increased risk of SNHL (all P<0.05). These working behaviors were also associated with increased severity of SNHL (all P<0.05) and a higher likelihood of bilateral SNHL (all P<0.05). In prospective studies, the trends were generally consistent with the aforementioned results. Furthermore, the relationship between night shift work and SNHL was particularly pronounced among individuals with morning chronotype (P-interaction=0.007), or with ≤5 years noisy work environments (P-interaction=0.026). Importantly, regardless of the level of genetic risk of PRS, a positive association remained between night shift work and physically demanding work with SNHL.

**Conclusions:**

Both cross-sectional and prospective analysis indicated that shift work, night shift work, and physically demanding work were associated with increased risk of occurrence, laterality and severity of SNHL, regardless of PRS for SHNL.

Work plays an indispensable role in the daily lives of most adults and is widely recognized as a determinant of physical function. Research has identified that engaging in physically demanding work is associated with a higher risk of various functional limitations and disability (1–3). One of the primary challenges associated with shift work is the disruption of the normal sleep–wake cycle, leading to reduced sleep duration and excessive fatigue (4). From a physiological perspective, shift work requires individuals to adhere to a sleep–wake schedule that generally conflicts with their natural endogenous rhythms of sleep and wakefulness, leading to circadian misalignment and the development of shift work disorder (SWD) (5).

According to the World Report on Hearing delivered by the World Health Organization in 2021, hearing loss is the most prevalent sensory impairment in humans, affecting more than 1.5 billion individuals worldwide. Sensorineural hearing loss (SNHL) refers to hearing loss caused by disorders of the cochlea, hearing nerve, or sometimes both, which is considered to be associated with aging, genetic mutation, noise exposure, ototoxic drugs, smoking, diabetes mellitus, autoimmune diseases and other risk factors (6). Disruption of circadian rhythms and sleep deprivation among shift and night workers have been associated with systemic physiological changes, including oxidative stress (7), immune dysregulation (8), and vascular damage (9), which may indirectly affect auditory function. Similarly, physically demanding work may increase exposure to occupational noise and contribute to vascular strain, which has been implicated in hearing loss (10).

In the past, studies have provided evidence that shift work was associated with hearing loss in specific occupational groups, such as retired employees of Dongfeng Motor Corporation (11) and male steel workers (12). However, no conclusive associations have been uncovered between the frequency of these working behaviors (shift work, night shift work and physically demanding work) and SNHL. Additionally, these studies have not shown any evidence indicating that these working behaviors have an impact on the laterality and severity of SNHL. Studies investigating the relationship between working behaviors and SNHL in a large natural population cohort are lacking.

In addition to environmental factors such as working behaviors, genetics also play an important role in the risk of SNHL. Currently, 115 genes have been identified to be responsible for non-syndromic hearing loss, including autosomal dominant genes, autosomal recessive genes, X-linked genes, and additional loci for modifiers, Y-linked, and auditory neuropathy (13). However, it is unclear whether genetic susceptibility affects the association between working behaviors and SNHL.

In this study, we conducted a cross-sectional study and a prospective study to investigate the potential association between current shift work, night shift work or physically demanding work and the occurrence (yes/no), laterality (unilateral/bilateral), and severity (mild/severe) of SNHL. Furthermore, by leveraging genetic information of study participants, we also explored whether genetic predisposition could affect the association between shift work, night shift work or physically demanding work and SNHL.

## Methods

### Study participants and design

UK Biobank (UKB) is a large-scale biomedical database and research resource, providing in-depth genetic and health information, including basic questionnaire, physical measures, health-related data and sample assays of over 500 000 adult participants (aged 40–69 years) recruited between 2006 and 2010 (baseline) and followed up until 2022.

We included participants who had speech-reception-threshold (SRT) estimate (left and right) test at baseline, were engaged in paid employment or self-employment, and completed a series of questionnaires about their work (N=94 868). Exclusion criteria encompassed the following: (i) participants diagnosed with ear-related conditions at baseline, including conductive hearing loss, disorders of middle ear, mastoid, vestibular function, inner ear, and external ear (N=724, supplementary material, www.sjweh.fi/article/4209, table S1); (ii) participants who were completely deaf (14) (either self-reported as “I am completely deaf” in questionnaires; or ICD9 code: 3897; or ICD10 code: H91.3) or deaf in one ear and did not have hearing assessment data for the other ear (N=3891); and (iii) participants withdrew from the project (N=75).

**Figure 1:**
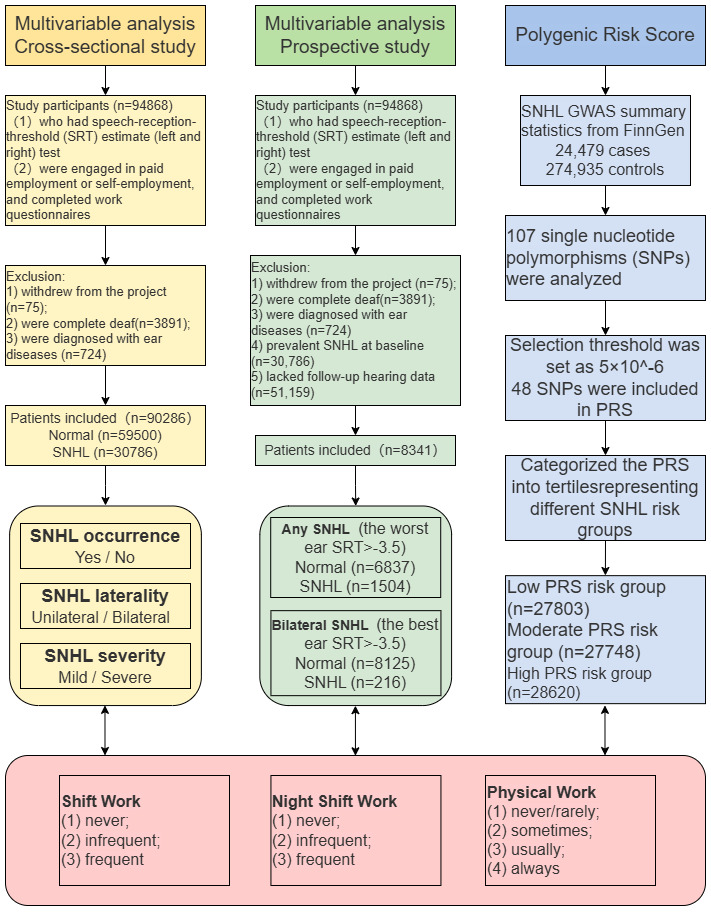
Flow chart of the study cohort selection.

To thoroughly investigate the relationship between working behaviors and SNHL, we conducted a comprehensive four-step analysis. Initially, a cross-sectional study was conducted encompassing all participants who fulfilled the designated criteria (N=90 286). Subsequently, we performed a prospective analysis by excluding individuals who exhibited prevalent SNHL at baseline (N=30 786) and those who lacked follow-up hearing test data (N=51 159). After rigorous screening, 8341 participants were included to assess the association between shift work, night shift work, physically demanding work, and new-onset SNHL. Furthermore, we delved into the relationship between working behaviors and the severity and laterality of SNHL. Finally, we performed a genetic analysis among participants of European descent with available genetic data to explore whether the polygenic risk score (PRS) could affect the association between working behaviors and SNHL. The flow chart of the study cohort selection is shown in figure 1.

### Working behaviors assessment

Currently working participants were asked whether their current job involved shift work. Those who answered affirmatively were further asked whether they participated in night shift work. Shift work was defined as a work schedule falling outside of the normal daytime working hours of 09:00–17:00 hours, potentially encompassing afternoon, evening or night shifts, or a rotation through these various shifts. Night shift work was defined as working during regular sleeping hours, for instance working through from 24:00–06:00 hours. Based on their self-reported responses in the questionnaires, participants were categorized into “never/rarely”, “infrequent” (participants sometimes involved shift/night shift work) and “frequent” (participants usually or always engaged in shift/night shift work).

Current working participants were also asked whether their work involved handling of heavy objects or tools. Participants were separated into “never/rarely”, “sometimes”, “usually” and “always” groups according to their answers. Individuals could belong to both physically demanding work group and shift/night shift work group.

### SNHL assessment

SNHL was identified through the SRT test, which was detected by the digit triplet test (DTT). Comprehensive descriptions of these procedures have been elaborated upon in our preceding publication (15). This diagnostic process involved a meticulous exclusion of ear-related conditions, including disorders of the middle ear, mastoid, vestibular function, inner ear, and external ear (supplementary table S1).

Unilateral SNHL was defined by the fulfillment of at least one of the following criteria: (i) one of the ears was tested for hearing loss (SRT ≥-5.5 dB) while the other was normal (SRT <-5.5 dB); and/or (ii) self-reported that “I can only hear on the left/right side” before DTT.

In the case of bilateral SNHL, the degree of hearing impairment was categorized as normal (SRT <-5.5 dB), mild (−5.5 dB– -3.5 dB), and severe (SRT >-3.5 dB) of the best ear (16). To account for both unilateral and bilateral SNHL in our analysis, we defined an outcome category termed “any SNHL” as the worst ear SRT ≥-5.5 dB, and any severe SNHL was defined as the worst ear SRT >-3.5 dB.

### Polygenic risk score for SNHL

Centralized analysis of the genetic data from UKB, including genotype quality, properties of population structure and relatedness of the genetic data, and efficient phasing and genotype imputation has been described earlier (17). We used the SNHL Genome-Wide Association Study (GWAS) summary statistics from FinnGen (r4.finngen.fi), which consisted of 24 479 cases and 274 935 controls, and about 107 single nucleotide polymorphisms (SNP) were analyzed. According to the effect values corresponding to the candidate SNP selected by GWAS, the selection threshold for SNP was set as 5×10^-6^ to consider the genetic predisposition for SNHL (supplementary table S2). Finally, 48 SNP were included in the calculation of PRS. The participants were stratified into three equal groups based on tertiles of genetic risk (low, moderate and high) to evaluate the association between working behaviors and SNHL in participants with different genetic predisposition.

### Covariates

We took into account a spectrum of demographic, lifestyle, and pre-existing comorbidities as covariates to ensure a robust assessment. Demographic information encompassed age, sex, body mass index (BMI), the Townsend deprivation index, years of education and ethnic background. Lifestyle information contained smoking status, alcohol drinker status, coffee intake, tea intake, regular physical activity and exposure to ototoxic medications, which was identified on the basis of self-reported regular use of ototoxic medications (including loop diuretics, aminoglycoside antibiotics, quinine derivatives, non-steroidal anti-inflammatories and salicylates) (18). In addition, years of exposure to noisy work environments, sleep duration and sleeplessness status were also included. In the fully adjusted model, we also accounted for pre-existing comorbidities that could influence the relationship between working behaviors and SNHL. These comorbidities included depression, bipolar disorder, anxiety, vascular/heart problems, allergic or thrombotic disorders, diabetes and cancers.

### Statistics analyses

For baseline characteristics, categorical variables were presented in terms of frequencies and proportions. Continuous variables, on the other hand, were summarized as mean, standard deviation (SD), median, and range. For the cross-sectional component of the study, multivariable logistic regression analyses were employed to assess the associations between working behaviors and SNHL. The results were articulately conveyed through odds ratios (OR) and 95% confidence intervals (CI). Regarding the prospective aspect of the research, multivariable Cox proportional hazards regression analyses were utilized. These findings were presented as relative risk (RR) and 95% CI. Both the logistic and Cox regression models were meticulously adjusted for a wide array of factors, including demographic information, lifestyle information and pre-existing comorbidities.

Subgroup analyses were also carried out to explore potential variations in the associations between working behaviors and SNHL across different participants’ characteristics, including sex (female/male), age (≤60 or >60 years), chronotype (morning, middle, evening) and noisy work environments (0, <1–5, and >5 years). To statistically assess the significance of the observed differences between these subgroups, a P-value of interaction was obtained through a likelihood ratio test, which compared the groups with and without interaction terms.

Additionally, we conducted a PRS analysis to investigate whether genetic susceptibility to SNHL modified the association between working behaviors, and the risk of developing SNHL. Initially, we verified the weighted PRS as a continuous variable and found that higher PRS values were positively associated with SNHL. Subsequently, to facilitate a more nuanced examination, we categorized the PRS into tertiles representing high, moderate, and low SNHL risk groups. By employing a log likelihood ratio test to compare models with and without cross-product interaction terms, we performed interaction analyses across various PRS categories.

All analyses were performed using R 4.0.4 (R Development Core Team, Vienna, Austria), and P value of <0.05 was considered statistically significant.

## Results

### Participants characteristics

The cross-sectional study included a total of 90 266 participants, with 74 322 individuals never engaged in shift work, 7668 only took part in day shift work, 4734 were occupied in infrequent night shift work and 3542 were involved in frequent night shift work (table 1). Furthermore, 36.1% of participants reported "never engaging in physically demanding work", while the remaining individuals engaged in varying degrees of physically demanding work.

**Table 1:** Clinical and demographic characteristics of all study subjects. (cross-sectional study) [SNHL: Sensorineural hearing Loss]

Variable		Never (N=74322)			Only day shift work (N=7668)			Infrequent night work (N=4734)			Frequent night work (N=3542)			All participants			P-value
		N (%)	Mean (SD)		N (%)	Mean (SD)		N (%)	Mean (SD)		N (%)	Mean (SD)		N (%)	Mean (SD)		
Physically demanding work																	<0.001
	Never	30 272 (40.8)			1224 (16)			672 (14.2)			435 (12.3)			32603 (36.1)			
	Sometimes	23004 (31)			2199 (28.7)			1455 (30.8)			794 (22.4)			27452 (30.4)			
	Usually	9741 (13.1)			1505 (19.7)			1049 (22.2)			823 (23.2)			13118 (14.5)			
	Always	11 259 (15.2)			2731 (35.7)			1550 (32.8)			1488 (42)			17028 (18.9)			
	Missing	46			9			8			2			65			
Sex																	<0.001
	Female	40 124 (54)			4061 (53)			1758 (37.1)			1363 (38.5)			47306 (52.4)			
	Male	34 198 (46)			3607 (47)			2976 (62.9)			2179 (61.5)			42960 (47.6)			
Age			53.1 (7.3)			52.6 (7.2)			51.3 (6.9)			51.3 (6.9)			52.9 (7.3)		<0.001
Body mass index																	<0.001
	Under or acceptable weight	26 604 (36)			2362 (31)			1206 (25.7)			883 (25.1)			31055 (34.6)			
	Obese	16 503 (22.3)			2045 (26.8)			1435 (30.6)			1096 (31.1)			21079 (23.5)			
	Overweight	30 840 (41.7)			3210 (42.1)			2051 (43.7)			1543 (43.8)			37644 (41.9)			
	Missing	375			51			42			20			488			
Ethnicity																	<0.001
	Non-white	5719 (7.7)			1144 (15)			789 (16.7)			654 (18.6)			8306 (9.2)			
	White	68 370 (92.3)			6492 (85)			3926 (83.3)			2868 (81.4)			81656 (90.8)			
	Missing	233			32			19			20			304			
Townsend deprivation index			-1.3 (2.8)			-0.4 (3)			-0.4 (3.1)			-0.3 (3.2)			-1.1 (2.9)		<0.001
	Missing	127			17			11			10			165			
Qualifications																	<0.001
	College or University degree	31 492 (42.6)			2014 (26.5)			1182 (25.2)			609 (17.4)			35297 (39.3)			
	Other	42 454 (57.4)			5585 (73.5)			3503 (74.8)			2889 (82.6)			54431 (60.7)			
	Missing	376			69			49			44			538			
Smoking status																	<0.001
	Never	43 581 (58.8)			4211 (55.1)			2531 (53.6)			1881 (53.3)			52204 (58)			
	Current	6912 (9.3)			1019 (13.3)			750 (15.9)			606 (17.2)			9287 (10.3)			
	Previous	23 662 (31.9)			2407 (31.5)			1437 (30.5)			1041 (29.5)			28547 (31.7)			
	Missing	167			31			16			14			228			
Alcohol status																	<0.001
	Never	2551 (3.4)			442 (5.8)			251 (5.3)			237 (6.7)			3481 (3.9)			
	Current	69 762 (93.9)			6928 (90.5)			4316 (91.3)			3143 (88.9)			84149 (93.3)			
	Previous	1966 (2.6)			288 (3.8)			162 (3.4)			157 (4.4)			2573 (2.9)			
	Missing	43			10			5			5			63			
Coffee intake			2 (2.1)			2 (2.2)			2.1 (2.3)			2.2 (2.4)			2 (2.1)		<0.001
	Missing	66			30			22			21			139			
Tea intake			3.3 (2.8)			3.5 (3.1)			3.6 (3.1)			3.8 (3.8)			3.4 (2.9)		<0.001
	Missing	73			18			20			22			133			
Total Metabolic Equivalent Task (MET) minutes			2523 (2647.9)			3290.7 (3251.4)			3502.1 (3374.1)			3643.1 (3400.6)			2683.5 (2801)		<0.001
Ototoxic medication																	<0.001
	No	57 370 (77.2)			5737 (74.8)			3529 (74.5)			2665 (75.2)			69301 (76.8)			
	Yes	16 952 (22.8)			1931 (25.2)			1205 (25.5)			877 (24.8)			20965 (23.2)			
Sleep duration																	<0.001
	7–8	52 194 (70.4)			4875 (63.8)			2811 (59.7)			1899 (54.2)			61779 (68.6)			
	<7	18 872 (25.4)			2374 (31.1)			1673 (35.5)			1392 (39.7)			24311 (27)			
	>8	3114 (4.2)			388 (5.1)			224 (4.8)			215 (6.1)			3941 (4.4)			
	Missing	142			31			26			36			235			
Sleeplessness																	0.002
	Never/rarely	20 785 (28)			1970 (25.7)			1308 (27.6)			1015 (28.8)			25078 (27.8)			
	Sometimes	35 102 (47.2)			3705 (48.4)			2268 (47.9)			1652 (46.8)			42727 (47.4)			
	Usually	18 410 (24.8)			1986 (25.9)			1155 (24.4)			860 (24.4)			22411 (24.8)			
	Missing	25			7			3			15			50			
Noisy workplace																	<0.001
	No	59 906 (81.2)			5381 (71.1)			2776 (59.6)			1992 (57.2)			70055 (78.3)			
	<1 year	4414 (6)			481 (6.4)			430 (9.2)			212 (6.1)			5537 (6.2)			
	>5 year	5886 (8)			1132 (15)			1009 (21.7)			930 (26.7)			8957 (10)			
	1–5 year	3540 (4.8)			569 (7.5)			442 (9.5)			350 (10)			4901 (5.5)			
	Missing	576			105			77			58			816			
Depression																	0.0194
	No	73 923 (99.5)			7607 (99.2)			4712 (99.5)			3526 (99.5)			89768 (99.4)			
	Yes	399 (0.5)			61 (0.8)			22 (0.5)			16 (0.5)			498 (0.6)			
Bipolar disorder																	0.4243
	No	74 260 (99.9)			7662 (99.9)			4733 (100)			3541 (100)			90196 (99.9)			
	Yes	62 (0.1)			6 (0.1)			1 (0)			1 (0)			70 (0.1)			
Anxiety																	0.8678
	No	74 162 (99.8)			7649 (99.8)			4724 (99.8)			3536 (99.8)			90071 (99.8)			
	Yes	160 (0.2)			19 (0.2)			10 (0.2)			6 (0.2)			195 (0.2)			
Vascular/heart problems (heart attack, angina, stroke, high blood pressure)																	<0.001
	No	57 632 (77.6)			5757 (75.3)			3511 (74.3)			2690 (76.1)			69590 (77.2)			
	Yes	16 613 (22.4)			1893 (24.7)			1212 (25.7)			847 (23.9)			20565 (22.8)			
	Missing	77			18			11			5			111			
Diabetes																	<0.001
	No	71 449 (96.3)			7263 (95.1)			4488 (95.1)			3321 (94.3)			86521 (96)			
	Yes	2754 (3.7)			377 (4.9)			233 (4.9)			200 (5.7)			3564 (4)			
	Missing	119			28			13			21			181			
Allergic/thrombus problems (blood clot in the leg/lung, emphysema/ chronic bronchitis, asthma, hay fever/ allergic rhinitis /eczema)																	<0.001
	No	49 354 (66.5)			5121 (66.9)			3250 (68.8)			2474 (70)			60199 (66.8)			
	Yes	24 911 (33.5)			2536 (33.1)			1477 (31.2)			1059 (30)			29983 (33.2)			
	Missing	57			11			7			9			84			
Cancer																	0.0012
	No	69 843 (94.2)			7197 (94.3)			4491 (95.1)			3361 (95.3)			84892 (94.3)			
	Yes	4331 (5.8)			438 (5.7)			229 (4.9)			164 (4.7)			5162 (5.7)			
	Missing	148			33			14			17			212			
SNHL																	<0.001
	No	49 682 (66.8)			4682 (61.1)			2974 (62.8)			2152 (60.8)			59490 (65.9)			
	Yes	24 640 (33.2)			2986 (38.9)			1760 (37.2)			1390 (39.2)			30776 (34.1)			
Job																	<0.001
	Managers and Senior Officials	14 651 (19.8)			997 (13)			517 (11)			219 (6.2)			16384 (18.2)			
	Professional Occupations	19 010 (25.6)			806 (10.5)			416 (8.8)			168 (4.8)			20400 (22.6)			
	Associated Profissional and Technical Occupations	12 530 (16.9)			1690 (22.1)			1382 (29.3)			961 (27.2)			16563 (18.4)			
	Administrative and Secretarial Occupations	12 542 (16.9)			707 (9.2)			172 (3.6)			182 (5.1)			13603 (15.1)			
	Skilled Trades Occupations	4799 (6.5)			638 (8.3)			566 (12)			301 (8.5)			6304 (7)			
	Personal Service Occupations	3679 (5)			837 (10.9)			597 (12.6)			550 (15.6)			5663 (6.3)			
	Sales and Customer Service Occupations	2135 (2.9)			636 (8.3)			118 (2.5)			104 (2.9)			2993 (3.3)			
	Process, Plant and Machine Operatives	2105 (2.8)			656 (8.6)			606 (12.8)			570 (16.1)			3937 (4.4)			
	Elementary Occupations	2729 (3.7)			688 (9)			347 (7.4)			481 (13.6)			4245 (4.7)			
	Missing	142			13			13			6			174			

In the prospective study, 8341 participants (any SNHL: N=1504; bilateral SNHL: N=216) were included (supplementary table S3). The majority of participants (86.6%) were classified as day workers, while others were shift or night shift workers. Moreover, 43.4% of participants reported never engaging in physically demanding work, 31.1% reported engaging sometimes, 13.1% reported usually, and 12.5% reported always being involved in physically demanding work (supplementary table S3).

### Cross-sectional study

**Figure 2:**
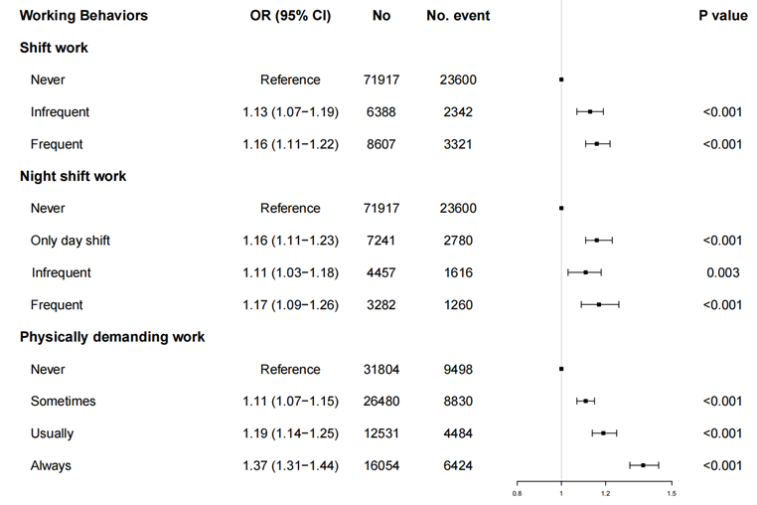
Association between shift work, night shift work, physically demanding work and any SNHL.

*The relationship between shift work, physically demanding work and occurrence of SNHL.* Our results showed that compared to day workers, shift work [including infrequent shift work (OR 1.13, 95% CI 1.07–1.19) and frequent shift work (OR 1.16, 95% CI 1.11–1.22)] were associated with an increased risk of SNHL. Consistent patterns were noted among infrequent night shift workers (OR 1.11, P=0.003) and frequent night shift workers (OR 1.17, P<0.001), both indicating a significant increase in SNHL risk compared to day workers. Notably, only day shift worker also indicated significant association with SNHL (OR 1.16, P<0.001). Furthermore, the level of physically demanding work engagement was found to be positively correlated with the odds of SNHL. Participants who reported engaging in physically demanding work sometimes (OR 1.11, P<0.001), usually (OR 1.19, P<0.001), and always (OR 1.37, P<0.001) had higher odds of SNHL compared to non-physical workers (table 2, figure 2). The results suggested a significant relationship between the frequency of shift work, night shift work, and physical work, and the risk of SNHL, which indicated that as the frequency of these working behaviors increased, the likelihood of SNHL also increased.

**Table 2:** Multivariable analysis ^a^ for shift work, night shift work, physical work associated with SNHL. [BMI=body mass index; CI=confidence interval; SNHL=sensorineural hearing loss].

Variable		N	Event	OR (95% CI)	P-value
Shift work					
	Never	71917	23 600	Reference	
	Infrequent	6388	2342	1.13 (1.07–1.19)	<0.001
	Frequent	8607	3321	1.16 (1.11–1.22)	<0.001
Night shift work					
	Never	71 917	23 600	Reference	
	Only day shift	7241	2780	1.16 (1.11–1.23)	<0.001
	Infrequent	4457	1616	1.11 (1.03–1.18)	0.003
	Frequent	3282	1260	1.17 (1.09–1.26)	<0.001
Physically demanding work					
	Never	31 804	9498	Reference	
	Sometimes	26 480	8830	1.11 (1.07–1.15)	<0.001
	Usually	12 531	4484	1.19 (1.14–1.25)	<0.001
	Always	16 054	6424	1.37 (1.31–1.44)	<0.001

^a^ Adjusted for age, sex, BMI, Townsend deprivation index, years of education and ethnic background, smoking status, alcohol drinker status, coffee intake, tea intake, regular physical activity, ototoxic medication intake, years of exposure to noisy work environments, sleep duration, sleeplessness status and pre-existing comorbidities.

*The relationship between shift work, physically demanding work and laterality of SNHL.* The multivariable analysis (Table S4 and Figure S1) delineated the association between working behaviors and the occurrence of unilateral and bilateral SNHL. The reference categories for these variables were individuals who never engaged in the respective work patterns.

Working behaviors demonstrated a clear dose-response relationship with SNHL, and the correlation is stronger with bilateral SNHL. The findings demonstrated that frequent shift work and night shift work emerged as significant risk factors for both unilateral SNHL [shift (OR 1.12, P<0.001) and night shift (OR 1.14, P=0.002)] and bilateral SNHL [shift (OR 1.35, P<0.001) and night shift (OR 1.33, P<0.001)]. Similarly, individuals who reported engaging in physically demanding work with varying frequencies showed a heightened probability of experiencing both unilateral SNHL [always (OR 1.29, P<0.001), usually (OR 1.15, P<0.001), and sometimes (OR 1.08, P<0.001)] and bilateral SNHL [always (OR 1.72, P<0.001); usually (OR 1.35, P<0.001), sometimes (OR 1.22, P<0.001)] compared to those who never engaged in physically demanding work .

Building on these observations, a multivariable analysis model was performed to evaluate the association between shift work, night shift work, physically demanding work, and bilateral SNHL in contrast to unilateral SNHL (supplementary table S5). The results revealed that both shift work and physically demanding work posed a higher risk for bilateral SNHL compared to unilateral SNHL.

*The relationship between shift work, physically demanding work and severity of SNHL.* When dividing participants with bilateral SNHL into mild and severe SNHL groups based on the hearing level of best ear, we observed that the risk of developing severe SNHL was higher among participants who had frequent shift work (OR 1.28, P<0.001) and frequent night shift work (OR 1.32, P<0.001). Only day shift workers also demonstrated significant association with mild SNHL (OR 1.11, P<0.001) and severe SNHL (OR 1.36, P<0.001). Moreover, the level of physically demanding work was found to be consistently linked to the risk of both mild and severe SNHL. Compared to the reference group, increased physically demanding work demonstrated a progressive association with the risk of mild SNHL, represented by OR of 1.08, 1.15, and 1.29, respectively (P<0.001), and severe SNHL, with OR of 1.18, 1.41, and 1.84, respectively (P<0.001), as detailed in supplementary table S6 and visualized in supplementary figure S2. These results implied that engaging in physically demanding work with greater frequency was correlated with an elevated risk of both mild and severe forms of SNHL. However, the increased frequency of shift/night shift work did not increase the risk of SNHL.

Additionally, it was observed that the influence of shift work and physically demanding work on the development of severe SNHL was markedly more pronounced than their impact on mild SNHL, as indicated in supplementary table S7.

### Subgroup analysis

The subgroup analysis examining the association between night shift work and SNHL is presented in supplementary table S8. After accounting for a multitude of variables, the relationship between night shift work and SNHL appeared to be influenced by chronotype (P-interaction=0.007). Specifically, night shift work was associated with an increased risk of SNHL among individuals with a morning chronotype [frequent (OR 1.32, P=0.001) and infrequent (OR 1.21, P=0.005)]. Furthermore, it was observed that the correlation between night shift work and the risk of SNHL was notably modulated by noisy work environments, as indicated by a significant P-interaction value of 0.026. Among individuals not exposed to noisy work environments or exposed for <1 year, both infrequent and frequent night shift work were significantly associated with an increased risk of SNHL. Interestingly, as the duration of exposure to noisy work environments increased, the effect of night shift work on SNHL risk diminished. Among individuals exposed to a noisy workplace for 1–5 years, the association with infrequent night shifts remained significant (OR 1.44, P<0.001), while the risk associated with frequent night shifts was not statistically significant (OR 1.21, P= 0.134). Among those exposed to a noisy workplace for >5 years, neither infrequent nor frequent night shift work demonstrated a significant association with SNHL risk. Additionally, the effect of night shift work on SNHL was consistent across sex (P-interaction =0.415) and age (P-interaction=0.427), suggesting that these factors did not significantly affect the relationship between night shift work and SNHL risk.

The subgroup analysis examining the potential association between physically demanding work and SNHL is shown in supplementary table S9. To explore the collective impact of various factors, interaction testing was performed to determine the combined influence of sex, age and noisy work environments. However, the results demonstrated that no such significant qualitative interactions were observed between physically demanding work and SNHL (P-interaction>0.05).

### Prospective study

In the prospective component of study, after the exclusion of individuals with pre-existing SNHL at baseline, there were 8341 participants included in the final analysis. Over the course of the follow-up period (until 2022), instances of new-onset SNHL were documented among the participants. The analysis revealed a statistically significant association between frequent shift work and the development of “any SNHL” (OR 1.24, P=0.029). Furthermore, a relationship was identified between the constant engagement in physically demanding work and the occurrence of bilateral SNHL (OR 1.60, P=0.028). Notably, participants who reported frequent night shift work exhibited a marked increase in the risk of developing “any SNHL” (OR 1.35, P=0.035) as well as bilateral SNHL (OR 1.98, P=0.044), as detailed in supplementary table S10. These findings underscored the potential long-term consequences of certain occupational exposures on hearing health and suggested that the risk of SNHL might escalate with the frequency of night shift work.

### Polygenic risk score

We investigated the potential influence of PRS on the relationship between shift work and the likelihood of developing SNHL among European participants (N=87 317). Initially, our findings indicated that an elevated genetic risk for SNHL, as measured by PRS, was correlated with higher odds of SNHL (OR 1.05, 95% CI 1.02–1.09, P=0.004) (supplementary table S11). Table 3 delineated the relationship between physically demanding work and the likelihood of SNHL across varying categories of genetic risk. Intriguingly, the positive correlation between SNHL and physically demanding work was observed to be consistent across all categories of PRS—low, moderate, and high. This suggested that physically demanding work might be a robust risk factor for SNHL, irrespective of an individual’s genetic predisposition. Furthermore, our analysis revealed a statistically significant interaction between the PRS for SNHL and the association with physically demanding work (P-interaction=0.038). However, the effect of this interaction did not appear to follow a linear pattern. The highest prevalence of SNHL was observed in individuals who were always engaged in physically demanding work and fell within the moderate PRS category (OR 1.44, 95% CI 1.33–1.57, P<0.001). In contrast, we did not detect any significant interaction between the PRS categories and night shift work (P-interaction=0.495), as further elaborated in supplementary table S12. This lack of interaction implied that the genetic risk, as quantified by PRS, might not significantly modify the relationship between night shift work and the risk of SNHL.

**Table 3:** Multivariable-adjusted odds ratios (OR) for weighted polygenic risk score (PRS), PRS tertiles and physical work. [CI=confidence interval.]

PRS	Physical work	N	Event	OR	P-value	P-interaction
Low	Never	9059	2640	Reference		0.038
	Sometimes	7625	2542	1.15(1.07–1.23)	<0.001	
	Usually	3512	1225	1.2(1.1–1.31)	<0.001	
	Always	4503	1724	1.34(1.23–1.46)	<0.001	
Moderate	Never	9141	2655	Reference		
	Sometimes	7572	2437	1.1(1.03–1.18)	0.005	
	Usually	3437	1287	1.35(1.24–1.47)	<0.001	
	Always	4480	1787	1.44(1.33–1.57)	<0.001	
High	Never	9229	2830	Reference		
	Sometimes	7692	2627	1.08(1.01–1.16)	0.021	
	Usually	3720	1338	1.13(1.04–1.23)	0.004	
	Always	4785	1986	1.38(1.27–1.5)	<0.001	

## Discussion

This large-scale study provides new insights into the relationship between working behaviors and the risk of SNHL. Several key findings emerged: (i) Shift work was associated with an increased risk of SNHL. Similarly, night shift work demonstrated the same trend. (ii) Physically demanding work was linked to higher odds of SNHL, with a progressive increase in risk as the frequency of physically demanding work increased. (iii) Both unilateral and bilateral SNHL risks escalated with the frequency of shift work, night shift work, and physically demanding work, with bilateral SNHL having a higher risk than unilateral SNHL. (iv) The association between working behaviors and SNHL was consistent across different degrees of SNHL, with severe SNHL showing a higher risk than mild SNHL. (v) Night shift work had a stronger effect on SNHL in individuals with a morning chronotype. (vi) The positive association between night shift work/physically demanding work and SNHL was consistent regardless of genetic susceptibility based on PRS.

A previous study illustrated that high physically demanding work demands markedly increased sickness absence and unemployment, thereby reducing working life expectancy (19). For instance, data from National Health Interview Survey showed currently-employed male miners suffered from increased prevalence of hearing loss compared to nonmanual labor industries workers (20). While noisy environments were usually blamed, our findings suggested that physically demanding work itself remains a risk factor for SNHL even after adjusting for noise exposure, suggesting other underlying factors to explore such as psychosocial stress and job strain (21). The observation that the correlation between night shift work and the risk of SNHL is modulated by noisy work environments, suggesting that the interaction between these two factors is complex and warrants further discussion. Previous research has demonstrated that prolonged exposure to noise significantly raises the risk of hearing loss (22, 23), particularly in the first 15 years (24). As a consequence, the detrimental effects of night shift work might be overshadowed by more immediate and severe impact of noise exposure, leading to a saturation effect where additional damage from night shifts becomes less pronounced.

Moreover, metabolic disorders, including dyslipidemia (25), decreasing platelets (26), hypercholesterolemia, low levels of coenzyme Q10 and nervonic acid (27) have been implicated in the development of SNHL, and these factors could potentially mediate the relationship between working behaviors and hearing loss.

Evidence demonstrated that the effect of shift work, including day and night shift work, was in connection with acute sleep loss (28). Insufficient sleep further caused adverse immunological and metabolic changes (29), thus increased the risk for chronic diseases (30). Exposure to artificial light during night shifts could further disrupt circadian rhythms (31), which are regulated by melatonin (32). Melatonin, a potent antioxidant, is believed to play a protective role against outer hair cell dysfunction, which is integral to hearing health (33). A deficiency in melatonin could therefore exacerbate the risk of developing SNHL (34).

Besides, alterations in sleep patterns and circadian misalignment have been shown to impact various cellular mechanisms and neurobehavioral processes. These disruptions are linked to conditions such as metabolic syndrome (35), overweight and obesity (36, 37), impaired glucose tolerance (38), cardiovascular disease (39), dyslipidemia (40), and hypertension (41). These health conditions are also associated with a higher rate of hearing deterioration (42). In our study, even after controlling for factors such as BMI, sleep duration, sleeplessness status and pre-existing cardiovascular conditions, the significant association between shift work and SNHL remained. This finding suggests that other potential mediators between shift work and SNHL need further investigation.

The frequency of physically demanding work and shift/night shift work also appeared to be proportional to the severity of SNHL, with these working behaviors more likely to cause bilateral SNHL. This finding suggested that these labor conditions may exacerbate the progression of SNHL, highlighting the need for regular hearing assessments in populations engaged in such work.

Interestingly, night shift workers with a morning chronotype were at a higher risk of SNHL. Morning chronotypes generally struggled more with circadian misalignment during night shifts, which may explain their increased vulnerability. This finding was consistent with previous research suggesting that morning chronotypes have lower adaptation scores for night shift work compared to evening chronotypes (43).

To explore whether genetic predisposition influences the relationship between working behaviors and SNHL, we constructed a PRS. Contrary to expectations of a linear relationship, we found that the effect of physically demanding work on SNHL was most pronounced among individuals with moderate genetic risk. This unexpected finding suggested that genetic susceptibility may interact with environmental factors in complex ways, warranting further investigation.

Despite the novel insights provided by our study, there are some limitations. First, the UKB cohort, being long-term and large-scale, may experience participant dropouts, potentially affecting the study’s prospective nature. Second, our research focused on currently employed individuals, excluding those who had retired, which may limit the generalizability of our findings. Third, the UKB cohort predominantly consisted of participants of European descent, limiting the applicability of our results to other ethnic or racial groups. Fourth, while we took care to exclude participants with ear-related conditions, there may still be other unmeasured factors that could influence the accuracy of our findings. Finally, the UKB categorizes shift work by self-report questionnaires, which may vary in interpretation among individual respondents.

In conclusion, our study suggests shift work, night shift work and physically demanding work are associated with the occurrence, laterality, and severity of SNHL, and that these associations persist across all levels of genetic risk. Individuals with a morning chronotype may be particularly vulnerable, highlighting the need for targeted interventions in this population.

## Supplementary material

Supplementary material
