# Supplementary material for: Working behaviors and the risk of sensorineural hearing loss: A large cohort study
Source: Scand J Work Environ Health. 2025 Feb 28;51(2):77–88. doi: 10.5271/sjweh.4209 (PMC11892729; doi:10.5271/sjweh.4209)

Working behaviors and the risk of sensorineural hearing loss: A large cohort study[1]

by Wendu Pang, MD, Yao Song, MD, Jun Xie, MD, Xiaohong Yan, MD, Yaxin Luo, PMH, Ke Qiu, MD, Yufang Rao, PhD, Di Deng, PhD, Minzi Mao, PhD, Junhong Li, MD, Danni Cheng, PhD, Wei Xu, PhD, Jianjun Ren, MD, PhD, Yu Zhao, MD, PhD [2]

1. Supplementary material
2. Correspondence to: Yu Zhao, Department of Oto-Rhino-Laryngology, West China Hospital, Sichuan University, No. 37 Guo Xue Alley, Chengdu, China. [E-mail: yuzhao@wchscu.edu.cn]

Table S1 Diagnosis of ear diseases for exclusion.

Otitis externa	ICD10 H60
Other disorders of external ear	ICD10 H61
Perichondritis of external ear	ICD10 H61.0
Impacted cerumen	ICD10 H61.2
Acquired stenosis of external ear canal	ICD10 H61.3
Other specified disorders of external ear	ICD10 H61.8
Disorder of external ear, unspecified	ICD10 H61.9
Disorders of external ear in diseases classified elsewhere	ICD10 H62
Otitis media	ICD10 H65<br>ICD10 H66<br>ICD10 H67
Otalgia and effusion of ear	ICD10 H92
Eustachian salpingitis and obstruction	ICD10 H68
Other disorders of eustachian tube	ICD10 H69
Cholesteatoma of middle ear	ICD10 H71
Mastoiditis and related conditions	ICD10 H70
Perforation of tympanic membrane	ICD10 H72
Other disorders of tympanic membrane	ICD10 H73
Other disorders of middle ear and mastoid	ICD10 H73
Other disorders of middle ear and mastoid in diseases classified elsewhere	ICD10 H75
Otosclerosis	ICD10 H80
Disorders of vestibular function	ICD10 H81
Vertiginous syndromes in diseases classified elsewhere	ICD10 H82
Other diseases of inner ear	ICD10 H83
Other disorders of ear, not elsewhere classified	ICD10 H93
Other disorders of ear in diseases classified elsewhere	ICD10 H94
Postprocedural disorders of ear and mastoid process, not elsewhere classified	ICD10 H95
Conductive hearing loss, bilateral	ICD10 H90.0
Conductive hearing loss, unilateral with unrestricted hearing on the contralateral side	ICD10 H90.1
Conductive hearing loss, unspecified	ICD10 H90.2
Mixed conductive and sensorineural hearing loss, bilateral	ICD10 H90.6
Mixed conductive and sensorineural hearing loss, unilateral with unrestricted hearing on the contralateral side	ICD10 H90.7

Mixed conductive and sensorineural hearing loss, unspecified	ICD10 H90.8
Ototoxic hearing loss	ICD10 H91.0
Congenital malformations of ear causing impairment of hearing	Q16
Disorders of external ear	380
Nonsuppurative otitis media and eustachian tube disorders	381
Suppurative and unspecified otitis media	382
Mastoiditis and related conditions	383
Other disorders of tympanic membrane	384
Other disorders of middle ear and mastoid	385
Vertiginous syndromes and other disorders of vestibular system	386
Otosclerosis	387
Other disorders of ear	388
Deafness	389

Table S2 Individual significant SNHL SNP. *SNHL: Sensorineural hearing Loss.*

**SNP**	**CHR**	**POS**	**Beta**	**SE**	***P-value***	**EA**	**NEA**
rs35096827	3	150616348	0.2345	0.0494	2.05E-06	T	C
rs12110409	6	25161761	-0.1568	0.0331	2.13E-06	C	T
rs111692650	7	97183665	0.2666	0.052	2.88E-07	T	C
rs112963117	7	16897907	0.6717	0.1177	1.16E-08	T	C
rs28369763	9	97367392	-0.376	0.078	1.43E-06	T	C
rs118000080	9	8590597	0.8979	0.1957	4.48E-06	C	T
rs12801002	11	67968159	3.606	0.7586	2.00E-06	C	T
rs887306	12	4696801	-0.144	0.0313	4.27E-06	A	G
rs111602397	14	38104327	0.4982	0.1072	3.34E-06	A	T
rs59925400	15	90061400	0.1234	0.0261	2.31E-06	T	C
rs16954293	16	55144195	-0.2613	0.0571	4.71E-06	A	G
rs72828360	16	82520226	0.4276	0.0931	4.39E-06	A	G
rs118079176	18	50571586	0.249	0.0507	9.02E-07	T	C
rs12953985	18	19790078	0.1289	0.0272	2.21E-06	A	G
rs13058269	22	48530074	0.2813	0.06	2.78E-06	G	T
rs587620443	1	144023991	-0.2945	0.0621	2.12E-06	C	G
rs6732760	2	11101112	-0.0621	0.0129	1.60E-06	G	A
rs10528	2	54889149	0.1062	0.0186	1.16E-08	C	T
rs143848409	3	116193370	0.2055	0.0414	6.97E-07	T	C
rs62401780	5	143755933	0.1652	0.0357	3.69E-06	A	G
rs4704097	5	73069581	0.0704	0.0127	2.98E-08	G	A
rs151244510	6	74134014	0.1377	0.0281	9.19E-07	A	G

rs112266013	6	15230743	0.0875	0.0189	3.55E-06	A	G
rs115596275	6	32420218	0.21	0.0221	1.73E-21	C	G
rs9493627	6	133789728	0.0735	0.0145	4.22E-07	A	G
rs3131621	6	31425499	0.0699	0.0141	7.10E-07	G	A
rs276695	6	76469115	0.0832	0.0144	8.01E-09	C	T
rs1365889	7	33992983	0.1113	0.0197	1.70E-08	T	C
rs4286873	7	103015677	0.1094	0.0223	9.23E-07	A	G
rs7826175	8	74259289	-0.0659	0.0129	3.53E-07	A	G
rs525817	8	103993578	-0.0645	0.0128	4.86E-07	C	T
rs4837295	9	131470972	0.1156	0.0216	8.37E-08	G	A
rs10901863	10	126812270	0.1002	0.014	7.79E-13	T	C
rs189961447	11	104289943	0.2014	0.0382	1.36E-07	A	T
rs61889475	11	22854789	-0.0696	0.0126	3.67E-08	G	T
rs77751702	13	20751053	0.1715	0.0344	6.05E-07	T	C
rs34876558	14	100597169	0.1498	0.0321	2.95E-06	A	G
rs55737225	15	89251311	-0.0717	0.0142	4.46E-07	T	C
rs188029302	15	35969508	0.8708	0.1694	2.75E-07	A	G
rs12591997	15	51681073	0.1332	0.0263	4.09E-07	A	C
rs60142697	16	57678900	0.0641	0.0139	3.87E-06	G	A
rs78421216	16	25509854	0.194	0.0413	2.65E-06	T	A
rs8090563	18	6678715	0.0736	0.0127	7.07E-09	A	T
rs2392794	19	472149	-0.0934	0.0163	1.07E-08	T	C
rs143450113	19	42714797	0.1552	0.0333	3.10E-06	T	C
rs146891756	20	1819541	-0.2576	0.0524	8.71E-07	T	C
rs1970546	20	59853938	-0.0642	0.0135	2.10E-06	G	A
rs6000881	22	38154544	0.0856	0.0129	3.05E-11	A	G

Table S3 Clinical and demographic characteristics of all study subjects. (prospective study) *SNHL: Sensorineural hearing Loss*

**Variable**	**Never (n=49682)**	**Only day shift (n=4682)**	**Infrequent (n=2974)**	**Frequent (n=2152)**	**All participants**	**P-value**
**Any SNHL (the worst ear SRT>-3.5)**						0.0936
No	5947(82.3)	411(79.8)	280(81.4)	199(77.1)	6837(82)	
Yes	1277(17.7)	104(20.2)	64(18.6)	59(22.9)	1504(18)	
Missing	42458	4167	2630	1894	51149	
**Bilateral SNHL (the best ear SRT>-3.5)**						0.5231
No	7043(97.5)	500(97.1)	334(97.1)	248(96.1)	8125(97.4)	
Yes	181(2.5)	15(2.9)	10(2.9)	10(3.9)	216(2.6)	
Missing	42458	4167	2630	1894	51149	
**Sex**						<0.001
Female	26769(53.9)	2479(52.9)	1073(36.1)	808(37.5)	31129(52.3)	
Male	22913(46.1)	2203(47.1)	1901(63.9)	1344(62.5)	28361(47.7)	
**Age**						<0.001
Mean(SD)	52.3(7.1)	51.7(7)	50.5(6.6)	50.4(6.5)	52.1(7.1)	
Median[Min,Max]	52[39, 70]	51[40, 70]	50[40, 69]	50[40, 70]	52[39, 70]	
**Body mass index**						<0.001
Under or acceptable weight	17876(36.1)	1467(31.5)	776(26.3)	530(24.7)	20649(34.9)	
Obesity	10945(22.1)	1222(26.2)	874(29.6)	654(30.5)	13695(23.1)	
Overweight	20657(41.7)	1971(42.3)	1305(44.2)	961(44.8)	24894(42)	
Missing	204	22	19	7	252	
**Ethnicity**						<0.001
Non-white	2870(5.8)	463(9.9)	324(10.9)	239(11.2)	3896(6.6)	
White	46675(94.2)	4195(90.1)	2640(89.1)	1901(88.8)	55411(93.4)	
Missing	137	24	10	12	183	
**Townsend deprivation index**						<0.001
Mean(SD)	-1.4(2.7)	-0.6(3)	-0.7(3)	-0.6(3.1)	-1.3(2.8)	
Median [Min, Max]	-1.9[-6.3, 9.9]	-1.2[-6.2, 8.9]	-1.4[-6.3, 9.1]	-1.2[-6.3, 8.7]	-1.8[-6.3, 9.9]	
Missing	83	8	6	7	104	
**Qualifications**						<0.001
College or University degree	21685(43.8)	1243(26.7)	744(25.2)	347(16.2)	24019(40.6)	
Other	27794(56.2)	3407(73.3)	2209(74.8)	1790(83.8)	35200(59.4)	
Missing	203	32	21	15	271	
**Smoking status**						<0.001

Never	29259(59)	2587(55.4)	1580(53.3)	1110(51.8)	34536(58.2)	
Current	4538(9.2)	641(13.7)	479(16.1)	379(17.7)	6037(10.2)	
Previous	15797(31.9)	1439(30.8)	908(30.6)	654(30.5)	18798(31.7)	
Missing	88	15	7	9	119	
**Alcohol status**						<0.001
Never	1342(2.7)	172(3.7)	113(3.8)	95(4.4)	1722(2.9)	
Current	47071(94.8)	4337(92.7)	2760(92.9)	1970(91.5)	56138(94.4)	
Previous	1245(2.5)	170(3.6)	99(3.3)	87(4)	1601(2.7)	
Missing	24	3	2	0	29	
**Coffee intake**						<0.001
Mean(SD)	2(2)	2.1(2.3)	2.2(2.4)	2.3(2.5)	2.1(2.1)	
Median [Min, Max]	2[0, 42]	1[0, 36]	2[0, 30]	2[0, 21]	2[0, 42]	
Missing	35	17	12	10	74	
**Tea intake**						<0.001
Mean (SD)	3.3(2.7)	3.5(3.2)	3.6(3.1)	3.8(3.4)	3.3(2.8)	
Median [Min, Max]	3[0, 42]	3[0, 50]	3[0, 35]	3[0, 40]	3[0, 50]	
Missing	40	12	9	9	70	
**Total Metabolic Equivalent Task (MET) minutes**						<0.001
Mean (SD)	2475.5(2580.8)	3300.4(3237.4)	3521.4(3289.4)	3649.3(3331.2)	2635.2(2731.6)	
Median [Min, Max]	1653[0, 19278]	2180.8[0, 19278]	2462[0, 19278]	2697.2[0, 19278]	1740[0, 19278]	
**Ototoxic medication**						<0.001
No	38516(77.5)	3487(74.5)	2233(75.1)	1633(75.9)	45869(77.1)	
Yes	11166(22.5)	1195(25.5)	741(24.9)	519(24.1)	13621(22.9)	
**Sleep duration**						<0.001
7-8	35249(71.1)	3017(64.7)	1781(60.1)	1189(55.8)	41236(69.5)	
<7	12382(25)	1406(30.2)	1032(34.8)	812(38.1)	15632(26.3)	
>8	1974(4)	237(5.1)	152(5.1)	129(6.1)	2492(4.2)	
Missing	77	22	9	22	130	
**Sleeplessness**						0.0018
Never/rarely	13947(28.1)	1188(25.4)	820(27.6)	597(27.8)	16552(27.8)	
Sometimes	23549(47.4)	2260(48.3)	1453(48.9)	1005(46.9)	28267(47.5)	
Usually	12174(24.5)	1234(26.4)	700(23.5)	542(25.3)	14650(24.6)	
Missing	12	0	1	8	21	
**Noisy workplace**						<0.001

No	40401(81.9)	3328(71.9)	1782(60.8)	1194(56.4)	46705(79.1)	
<1 year	3019(6.1)	291(6.3)	274(9.3)	132(6.2)	3716(6.3)	
>5 year	3612(7.3)	656(14.2)	623(21.2)	581(27.4)	5472(9.3)	
1-5 year	2320(4.7)	356(7.7)	253(8.6)	211(10)	3140(5.3)	
Missing	330	51	42	34	457	
**Depression**						0.0047
No	49437(99.5)	4641(99.1)	2962(99.6)	2140(99.4)	59180(99.5)	
Yes	245(0.5)	41(0.9)	12(0.4)	12(0.6)	310(0.5)	
**Bipolar disorder**						0.5222
No	49644(99.9)	4677(99.9)	2973(100)	2152(100)	59446(99.9)	
Yes	38(0.1)	5(0.1)	1(0)	0(0)	44(0.1)	
**Anxiety**						0.8011
No	49566(99.8)	4674(99.8)	2966(99.7)	2148(99.8)	59354(99.8)	
Yes	116(0.2)	8(0.2)	8(0.3)	4(0.2)	136(0.2)	
**Vascular/heart problems**						<0.001
No	39251(79.1)	3616(77.3)	2274(76.6)	1683(78.3)	46824(78.8)	
Yes	10390(20.9)	1060(22.7)	696(23.4)	466(21.7)	12612(21.2)	
Missing	41	6	4	3	54	
**Diabetes**						<0.001
No	48030(96.8)	4465(95.7)	2842(95.8)	2052(95.7)	57389(96.6)	
Yes	1590(3.2)	202(4.3)	125(4.2)	92(4.3)	2009(3.4)	
Missing	62	15	7	8	92	
**Allergic/thrombus problems**						0.0011
No	32715(65.9)	3108(66.4)	1992(67.1)	1500(69.9)	39315(66.1)	
Yes	16935(34.1)	1571(33.6)	978(32.9)	647(30.1)	20131(33.9)	
Missing	32	3	4	5	44	
**Cancer**						0.0107
No	46798(94.4)	4418(94.7)	2838(95.6)	2043(95.4)	56097(94.5)	
Yes	2784(5.6)	247(5.3)	132(4.4)	99(4.6)	3262(5.5)	
Missing	100	17	4	10	131	

Table S4 Multivariable analysis for shift work, night shift work, physically demanding work associated with Unilateral and Bilateral SNHL. *SNHL: Sensorineural hearing Loss.*

**Variable**	**Unilateral SNHL**				**Bilateral SNHL**			
	**N**	**Event**	**OR (95% CI)**	**P-value**	**N**	**Event**	**OR (95% CI)**	**P-value**
**Shift work**								
Never	66306	17989	Reference		53928	5611	Reference	
Infrequent	5745	1699	1.1 (1.03-1.16)	**0.003**	4689	643	1.23 (1.12-1.35)	**<0.001**
Frequent	7610	2324	1.12 (1.06-1.18)	**<0.001**	6283	997	1.35 (1.25-1.47)	**<0.001**
**Night shift work**								
Never	66306	17989	Reference		53928	5611	Reference	
Only day shift	6432	1971	1.12 (1.05-1.18)	**<0.001**	5270	809	1.32 (1.21-1.44)	**<0.001**
Infrequent	3992	1151	1.07 (0.99-1.15)	0.082	1783	231	1.16 (1-1.35)	**0.047**
Frequent	2918	896	1.14 (1.05-1.24)	**0.002**	3909	598	1.33 (1.2-1.47)	**<0.001**
**Physically demanding work**								
Never	29783	7477	Reference		24327	2021	Reference	
Sometimes	24316	6666	1.08 (1.04-1.12)	**<0.001**	19814	2164	1.22 (1.14-1.3)	**<0.001**
Usually	11355	3308	1.15 (1.09-1.21)	**<0.001**	9223	1176	1.35 (1.25-1.47)	**<0.001**
Always	14173	4543	1.29 (1.22-1.35)	**<0.001**	11511	1881	1.72 (1.59-1.86)	**<0.001**

Adjusted for age, sex, BMI, Townsend deprivation index, years of education and ethnic background, smoking status, alcohol drinker status, coffee intake, tea intake, regular physical activity, ototoxic medication intake, years of exposure to noisy work environments, sleep duration, sleeplessness status and pre-existing comorbidities.

Table S5 Multivariable analysis for shift work, night shift work, physically demanding work associated with Bilateral SNHL compared with Unilateral SNHL. *SNHL: Sensorineural hearing Loss.*

**Variable**	**N**	**Event**	**OR (95% CI)**	**P-value**
**Shift work**				
Never	23600	5611	Reference	
Infrequent	2342	643	1.15 (1.04-1.26)	**0.007**
Frequent	3321	997	1.19 (1.09-1.29)	**<0.001**
**Night shift work**				
Never	23600	5611	Reference	
Only day shift	2780	809	1.19 (1.09-1.31)	**<0.001**
Infrequent	1616	465	1.17 (1.04-1.31)	**0.009**
Frequent	1260	364	1.12 (0.98-1.28)	0.093
**Physically demanding work**				
Never	9498	2021	Reference	
Sometimes	8830	2164	1.11 (1.03-1.19)	**0.004**
Usually	4484	1176	1.18 (1.08-1.28)	**<0.001**
Always	6424	1881	1.29 (1.19-1.4)	**<0.001**

Adjusted for age, sex, BMI, Townsend deprivation index, years of education and ethnic background, smoking status, alcohol drinker status, coffee intake, tea intake, regular physical activity, ototoxic medication intake, years of exposure to noisy work environments, sleep duration, sleeplessness status and pre-existing comorbidities.

Table S6 Multivariable analysis for shift work, night shift work, physically demanding work associated with Mild and Severe SNHL. *SNHL: Sensorineural hearing Loss.*

**Variable**	**Mild SNHL**				**Severe SNHL**			
	**N**	**Event**	**OR (95% CI)**	**P-value**	**N**	**Event**	**OR (95% CI)**	**P-value**
**Shift work**								
Never	66743	18426	Reference		52994	4677	Reference	
Infrequent	7661	2375	1.11 (1.05-1.17)	**<0.001**	6145	859	1.4 (1.28-1.52)	**<0.001**
Frequent	5781	1735	1.09 (1.03-1.16)	**0.006**	4604	558	1.28 (1.16-1.42)	**<0.001**
**Night shift work**								
Never	66743	18426	Reference		52994	4677	Reference	
Only day shift	6479	2018	1.11 (1.05-1.18)	**<0.001**	5152	691	1.36 (1.24-1.49)	**<0.001**
Infrequent	4007	1166	1.05 (0.98-1.13)	0.167	3259	418	1.35 (1.2-1.51)	**<0.001**
Frequent	2942	920	1.14 (1.05-1.24)	**0.002**	2330	308	1.32 (1.16-1.51)	**<0.001**
**Physically demanding work**								
Never	29783	7477	Reference		23970	1664	Reference	
Sometimes	24316	6666	1.08 (1.04-1.12)	**<0.001**	19378	1728	1.18 (1.1-1.27)	**<0.001**
Usually	11355	3308	1.15 (1.09-1.21)	**<0.001**	9058	1011	1.41 (1.3-1.54)	**<0.001**
Always	14173	4543	1.29 (1.22-1.35)	**<0.001**	11314	1684	1.84 (1.69-2)	**<0.001**

Adjusted for age, sex, BMI, Townsend deprivation index, years of education and ethnic background, smoking status, alcohol drinker status, coffee intake, tea intake, regular physical activity, ototoxic medication intake, years of exposure to noisy work environments, sleep duration, sleeplessness status and pre-existing comorbidities.

Table S7 Multivariable analysis for shift work, night shift work, physically demanding work associated with Severe SNHL compared with Mild SNHL. *SNHL: Sensorineural hearing Loss.*

**Variable**	**N**	**Event**	**OR (95% CI)**	**P-value**
**Shift work**				
Never	23103	4677	Reference	
Infrequent	2293	558	1.19 (1.07-1.32)	**0.001**
Frequent	3234	859	1.22 (1.12-1.34)	**<0.001**
**Night shift work**				
Never	23103	4677	Reference	
Only day shift	2709	691	1.21 (1.1-1.34)	**<0.001**
Infrequent	1584	418	1.27 (1.12-1.43)	**<0.001**
Frequent	1228	308	1.12 (0.98-1.29)	0.099
**Physically demanding work**				
Never	9320	1664	Reference	
Sometimes	8633	1728	1.07 (0.99-1.16)	0.075
Usually	4392	1011	1.24 (1.13-1.36)	**<0.001**
Always	6258	1684	1.43 (1.31-1.56)	**<0.001**

Adjusted for age, sex, BMI, Townsend deprivation index, years of education and ethnic background, smoking status, alcohol drinker status, coffee intake, tea intake, regular physical activity, ototoxic medication intake, years of exposure to noisy work environments, sleep duration, sleeplessness status and pre-existing comorbidities.

Table S8 Association of frequency of night shift work and SNHL ORs in subgroup analyses.

Variable	Category	Night shift work	N	Event	OR (95% CI)	P-value	P-interaction
**Sex**	Female	Never	38888	12805	Reference		0.415
		Only day shift	3859	1489	1.2(1.12-1.29)	**<0.001**	
		Infrequent	1664	638	1.21(1.09-1.34)	**<0.001**	
		Frequent	1271	509	1.23(1.09-1.39)	**<0.001**	
	Male	Never	33029	10795	Reference		
		Only day shift	3382	1291	1.12(1.04-1.21)	**0.003**	
		Infrequent	2793	978	1.04(0.95-1.13)	0.417	
		Frequent	2011	751	1.13(1.02-1.24)	**0.019**	
**Age**	≤60	Never	58594	17439	Reference		0.427
		Only day shift	6046	2167	1.16(1.1-1.23)	**<0.001**	
		Infrequent	3946	1354	1.08(1.01-1.16)	**0.035**	
		Frequent	2895	1036	1.11(1.03-1.21)	**0.01**	
	>60	Never	13323	6161	Reference		
		Only day shift	1195	613	1.09(0.97-1.23)	0.151	
		Infrequent	511	262	1.03(0.86-1.24)	0.745	
		Frequent	387	224	1.26(1.02-1.56)	**0.032**	
**Chronotype**	Middle	Never	42186	13592	Reference		**0.007**
		Only day shift	4120	1501	1.1(1.03-1.18)	**0.005**	
		Infrequent	2493	884	1.09(1-1.19)	0.05	
		Frequent	1763	649	1.13(1.02-1.25)	**0.024**	
	Morning	Never	16861	5904	Reference		
		Only day shift	1876	830	1.36(1.23-1.51)	**<0.001**	
		Infrequent	1104	448	1.21(1.06-1.38)	**0.005**	
		Frequent	641	284	1.32(1.11-1.56)	**0.001**	
	Evening	Never	5921	1888	Reference		
		Only day shift	581	201	1.07(0.89-1.28)	0.49	
		Infrequent	413	121	0.9(0.72-1.14)	0.388	
		Frequent	471	185	1.27(1.03-1.56)	**0.022**	
**Noisy work environments**	No	Never	58512	18921	Reference		**0.026**
		Only day shift	5175	1955	1.18(1.11-1.25)	**<0.001**	
		Infrequent	2664	936	1.08(0.99-1.17)	0.087	
		Frequent	1874	739	1.23(1.11-1.36)	**<0.001**	

	<1 year	Never	4311	1352	Reference		
		Only day shift	462	182	1.34(1.09-1.65)	**0.005**	
		Infrequent	409	145	1.24(0.99-1.55)	0.062	
		Frequent	199	76	1.43(1.05-1.94)	**0.023**	
	1-5 year	Never	3429	1166	Reference		
		Only day shift	534	200	1.1(0.9-1.34)	0.363	
		Infrequent	426	181	1.44(1.16-1.79)	**<0.001**	
		Frequent	328	126	1.21(0.94-1.55)	0.134	
	>5 year	Never	5665	2161	Reference		
		Only day shift	1070	443	1.08(0.94-1.24)	0.308	
		Infrequent	958	354	1.01(0.87-1.17)	0.922	
		Frequent	881	319	1.01(0.86-1.18)	0.911	
**Physically demanding work**	No	Never	28591	8396	Reference		0.693
		Only day shift	1147	387	1.16(1.02-1.32)	**0.026**	
		Infrequent	628	210	1.2(1.01-1.43)	**0.039**	
		Frequent	398	139	1.23(0.99-1.53)	0.058	
	Yes	Never	41004	14341	Reference		
		Only day shift	5864	2299	1.13(1.06-1.2)	**<0.001**	
		Infrequent	3695	1348	1.05(0.98-1.13)	0.195	
		Frequent	2787	1080	1.13(1.03-1.22)	**0.006**	

Table S9 Association of frequency of physically demanding work and SNHL ORs in subgroup analyses.

Variable	Category	Physically demanding work	N	Event	OR (95% CI)	P-value	P-interaction
**Sex**	Female	Never	15683	4750	Reference		0.115
		Sometimes	12108	4103	1.12 (1.06-1.18)	**<0.001**	
		Usually	5669	2003	1.18 (1.1-1.26)	**<0.001**	
		Always	7575	3008	1.38 (1.29-1.47)	**<0.001**	
	Male	Never	12362	3612	Reference		
		Sometimes	11252	3691	1.1 (1.03-1.16)	**0.002**	
		Usually	5189	1908	1.24 (1.15-1.33)	**<0.001**	
		Always	6446	2607	1.39 (1.29-1.5)	**<0.001**	
**Age**	≤60	Never	23490	6403	Reference		0.788
		Sometimes	18913	5711	1.12 (1.07-1.17)	**<0.001**	
		Usually	8861	2916	1.24 (1.17-1.31)	**<0.001**	
		Always	11406	4239	1.42 (1.35-1.5)	**<0.001**	
	>60	Never	4555	1959	Reference		
		Sometimes	4447	2083	1.12 (1.03-1.21)	**0.013**	
		Usually	1997	995	1.22 (1.09-1.36)	**<0.001**	
		Always	2615	1376	1.3 (1.17-1.45)	**<0.001**	
**Noisy work environments**	No	Never	24701	7333	Reference		0.064
		Sometimes	18745	6256	1.12 (1.07-1.17)	**<0.001**	
		Usually	7700	2706	1.18 (1.11-1.25)	**<0.001**	
		Always	9133	3619	1.39 (1.31-1.47)	**<0.001**	
	< 1 year	Never	1455	422	Reference		
		Sometimes	1504	447	0.96 (0.81-1.13)	0.596	
		Usually	761	293	1.39 (1.14-1.69)	**0.001**	
		Always	900	353	1.37 (1.12-1.68)	**0.002**	
	1-5 years	Never	966	309	Reference		
		Sometimes	1827	664	1.23 (1.04-1.46)	**0.018**	
		Usually	1627	633	1.35 (1.13-1.61)	**<0.001**	
		Always	2894	1192	1.45 (1.22-1.71)	**<0.001**	
	>5 years	Never	923	298	Reference		
		Sometimes	1284	427	1 (0.83-1.21)	0.981	
		Usually	770	279	1.15 (0.93-1.43)	0.19	
		Always	1094	451	1.4 (1.14-1.72)	**0.002**	

Table S10 Multivariable analysis for shift work, night shift work, physically demanding work associated with any and Bilateral SNHL (Prospective Study). *SNHL: Sensorineural hearing Loss.*

	**Any SNHL (the worst ear SRT>-3.5)**				**Bilateral SNHL (the best ear SRT>-3.5)**			
**Variable**	**N**	**Event**	**RR (95% CI)**	**P-value**	**N**	**Event**	**RR (95% CI)**	**P-value**
**Shift work**								
Never	7075	1243	Reference		7075	174	Reference	
Infrequent	508	98	1.12 (0.91-1.38)	0.273	508	18	1.59 (0.97-2.6)	0.068
Frequent	584	126	1.24 (1.02-1.49)	**0.029**	584	17	1.27 (0.76-2.13)	0.355
**Night shift work**								
Never	7075	1243	Reference		7075	174	Reference	
Only day shift	507	104	1.15 (0.94-1.41)	0.176	507	15	1.21 (0.71-2.06)	0.481
Infrequent	338	64	1.12 (0.87-1.45)	0.374	338	10	1.43 (0.74-2.74)	0.284
Frequent	247	56	1.35 (1.02-1.77)	**0.035**	247	10	1.98 (1.02-3.83)	**0.044**
**Physically demanding work**								
Never	3561	619	Reference		3561	84	Reference	
Sometimes	2527	430	0.95 (0.84-1.08)	0.428	2527	54	0.87 (0.62-1.23)	0.428
Usually	1073	212	1.07 (0.91-1.25)	0.442	1073	31	1.18 (0.77-1.8)	0.451
Always	1002	206	1.07 (0.9-1.27)	0.454	1002	40	1.6 (1.05-2.42)	**0.028**

Adjusted for age, sex, BMI, Townsend deprivation index, years of education and ethnic background, smoking status, alcohol drinker status, coffee intake, tea intake, regular physical activity, ototoxic medication intake, years of exposure to noisy work environments, sleep duration, sleeplessness status and pre-existing comorbidities.

Table S11 Multivariable-adjusted ORs (95%CIs) for weighted PRS, PRS tertiles and SNHL

**Polygenic risk**	**N**	**Event**	**OR (95% CI)**	**P-value**
**Low PRS**	27803	9176	Reference	
**Moderate PRS**	27748	9198	1.00 (0.96-1.04)	0.984
**High PRS**	28620	9859	1.05 (1.02-1.09)	**0.004**

Table S12 Multivariable-adjusted ORs (95%CIs) for weighted PRS, PRS tertiles and night shift work

Polygenic risk	Night Shift work	N	Event	OR (95% CI)	P-value	P-interaction
**Low**	Never	23067	7425	Reference		0.495
	Only day shift	2273	845	1.14(1.04-1.26)	**0.005**	
	Infrequent	1435	515	1.18(1.05-1.32)	**0.006**	
	Frequent	1024	390	1.28(1.12-1.47)	**<0.001**	
**Moderate**	Never	22943	7404	Reference		
	Only day shift	2349	893	1.18(1.08-1.3)	**<0.001**	
	Infrequent	1407	511	1.13(1-1.27)	**0.048**	
	Frequent	1046	389	1.11(0.97-1.27)	0.125	
**High**	Never	23620	7929	Reference		
	Only day shift	2392	951	1.18(1.08-1.29)	**<0.001**	
	Infrequent	1485	534	1.02(0.91-1.14)	0.779	
	Frequent	1115	440	1.14(1-1.3)	0.052	

Figure S1 Association between shift work, night shift work, physically demanding work and unilateral or bilateral SNHL.

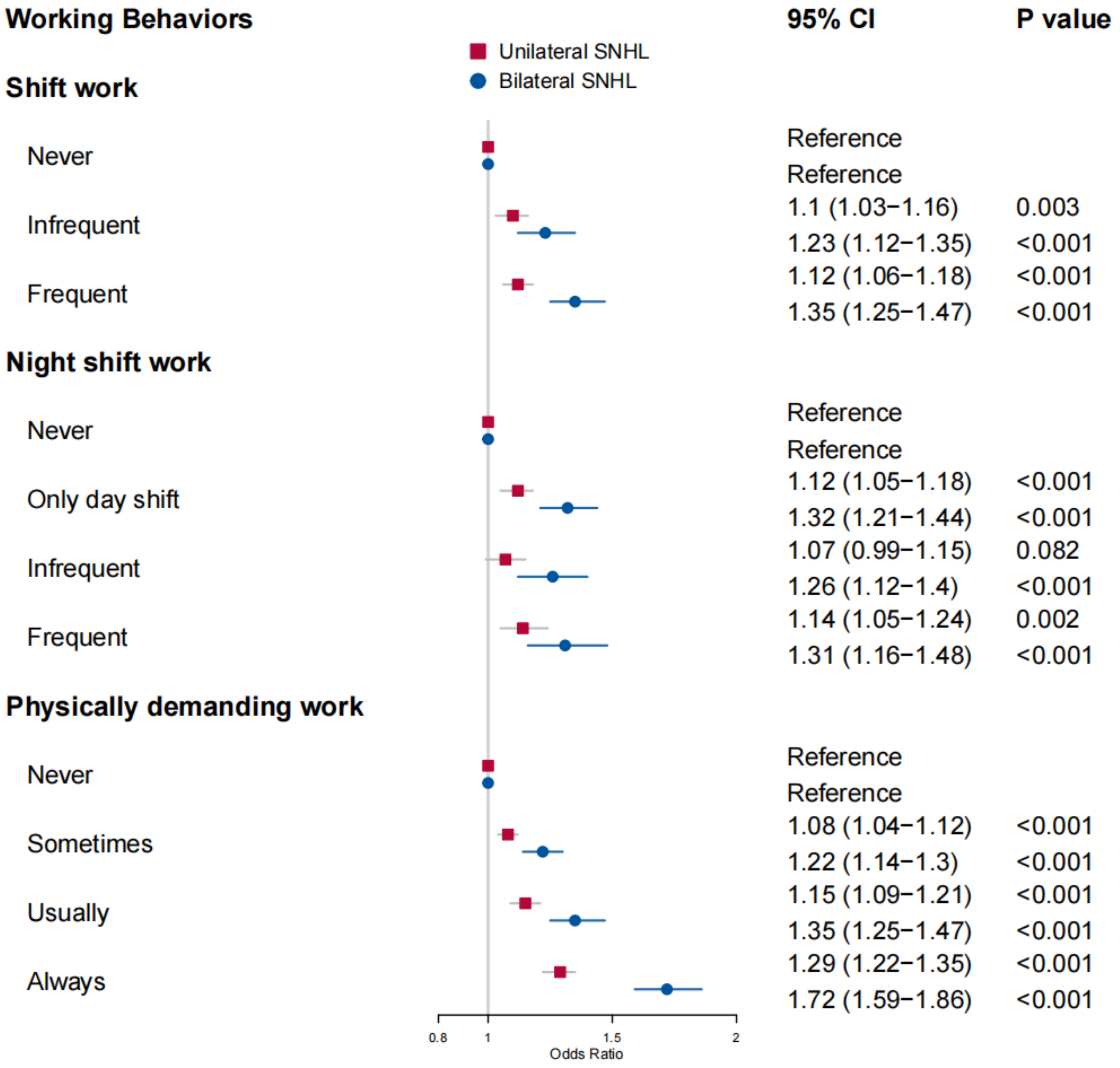

Figure S2 Association between shift work, night shift work, physically demanding work and mild or severe SNHL.

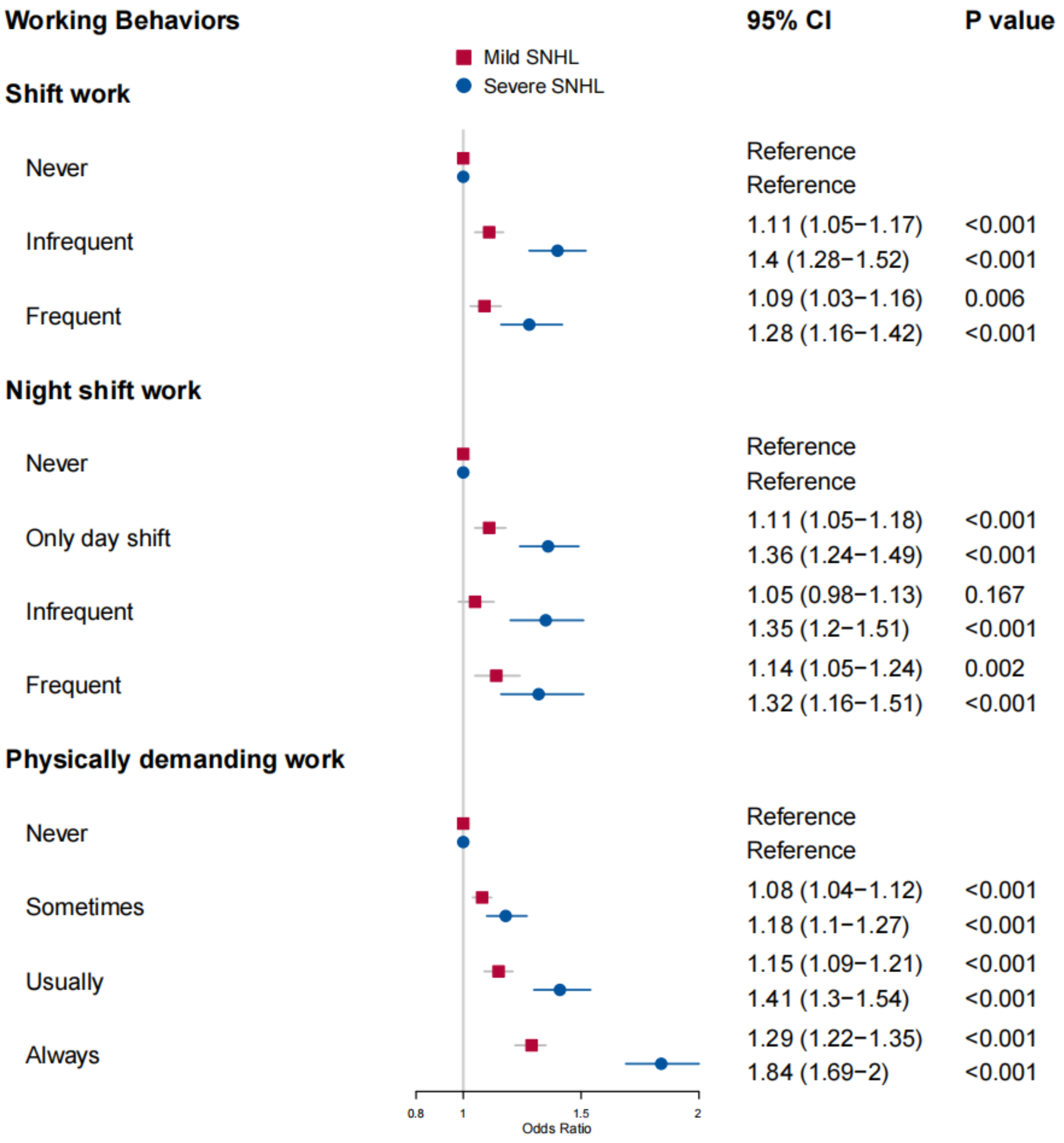

Supplement: Supplementary material [file SJWEH-51-77-S001.pdf]
